# Editorial: When predictions meet experiments: the future of structure determination

**DOI:** 10.3389/fmolb.2024.1446746

**Published:** 2024-07-19

**Authors:** Caterina Alfano

**Affiliations:** Structural Biology and Biophysics Unit, Fondazione Ri.MED, Palermo, Italy

**Keywords:** structural biology, AlphaFold, structure prediction, protein folding, machine-learning

Figuring out the “protein folding problem,” that is predicting protein structure from its amino acid sequence, has posed a significant challenge in biology for the past five decades. Thanks to the efforts of several generations of structural biologists, bioinformatics and artificial intelligence (AI) experts, in 2020 the organisers of the biennial Critical Assessment of protein Structure Prediction (CASP) competition heralded the AlphaFold program as a solution to this long-standing problem (Callaway, 2020).

This major breakthrough underscores the profound impact AI can have on scientific discovery and its potential to expedite advancements in fundamental research fields. The advent of AlphaFold in fact represents a paradigm shift that demands recognition and appreciation.

Nevertheless, the new developments have led to a misconception among some that experimental approaches to structure determination are now obsolete and unnecessary.

To address the divergence between these perspectives, an EMBO Workshop was organized in September 2022 in Palermo (Italy) aiming to foster dialogue on integrating AI predictions in the traditional practice of structure determination, enhancing the capabilities of both experimentalists and computational scientists. The theme of the present Research Topic, titled “When Predictions Meet Experiments: The Future of Structure Determination,” stems from the Palermo meeting and aims at comparing predictions and experiments in Structural Biology in the light of the recent breakthroughs linked to the advent of Alphafold.

The theme is well introduced by Carugo and Djinovic-Carugo who assert that protein structure prediction and structural biology have entered a new era with AI-driven approaches such as AlphaFold2 and RoseTTAfold methods. The authors assessed the extent to which these computational models can provide information on subtle structural details and focused on chalcogen bonds formed by disulfide bridges. Their findings indicate that only 43% of the chalcogen bonds observed in experimental structures are present in the computational models, suggesting that the accuracy of the computational models is, in the majority of cases, inadequate for detecting chalcogen bonds, according to the usual stereochemical criteria. Thus, high-resolution experimentally derived structures remain indispensable.

Additional contributions address specific aspects of structural biology in which the potential impact of AI was not immediately apparent. Ramakrishnan et al. noticed that predicting pathogenicity of missense variants in molecular diagnostics remains a challenge despite the availability of wealth data, such as evolutionary information, and the wealth of tools to integrate the data. They described DeepRank-Mut, a configurable framework designed to extract and learn from physico-chemically relevant features of amino acids surrounding missense variants in three-dimensional space. For each variant, various atomic and residue-level features are extracted from its structural environment, including sequence conservation scores of the surrounding amino acids, and stored in multi-channel 3D voxel grids which are than used to train a 3D convolutional neural network. The resultant model gives a probabilistic estimate of whether a given input variant is disease-causing or benign, highlighting considerations when adopting deep learning approaches for protein structure-guided pathogenicity predictions.

Perlinska et al. explored the intricate problem of protein knots, which have intrigued structural biologists for over three decades. Indeed, although most structural biologists have been aware of the existence of knotted proteins, it is hard to predict what is the most complicated knot that can be formed in proteins. Here, the authors show the most complex knotted topologies recorded to date, i.e., double trefoil knots (3 1 #3 1). They found five domain arrangements that result in a doubly knotted structure in over almost a thousand proteins. The double knot topology is found in knotted membrane proteins from the CaCA family, which function as ion transporters, in the group of carbonic anhydrases that catalyze the hydration of carbon dioxide, and in the proteins from the SPOUT superfamily that gathers 3 1 knotted methyltransferases with the active site-forming knot. For each family, they predicted the presence of a double knot using AlphaFold and RoseTTaFold structure prediction. In the case of the TrmD-Tm1570 protein, which is a member of the SPOUT superfamily, the authors showed that it folds *in vitro* and is biologically active. Their results show that this protein forms a homodimeric structure and retains the ability to modify tRNA, which is the function of the single-domain TrmD protein. However, how the protein folds and is degraded remains unknown.

In the fourth Research Topic Wetton et al. proposed a deep-learning-based workflow for NMR spectroscopy, ARTINA-CST, that automates the procedure for chemical shift transfer (CST). This is a well-established NMR technique that utilizes the chemical shift assignment of one protein to identify chemical shifts of another. The tool developed by Wetton et al. allows CST to be carried out within minutes or hours of computational time and strictly without any human supervision. Given its potential applications spanning a wide range of NMR projects, including drug discovery and protein interaction studies, ARTINA-CST holds the promise to be a valuable method that facilitates research in the field.

Finally, Dudas et al. integrated both experimental data and computer-assisted structure prediction tools to characterize annexin 11 (ANXA11), a calcium-dependent phospholipid-binding protein belonging to the annexin protein family and implicated in the neurodegenerative amyotrophic lateral sclerosis. Structurally, ANXA11 contains a conserved calcium-binding C-terminal domain common to all annexins and a putative intrinsically unfolded N-terminus specific for ANXA11. Little is known about the structure and functions of this region of the protein. The authors studied the structural features of the full-length protein with special attention to the N-terminal region using a combination of computational and biophysical techniques which include NMR and small angle X-ray scattering. Their work paves the way to a more thorough understanding of the ANXA11 functions and represents how AI predictions can be integrated into the normal practice of structure determination to increase the capabilities of both experimentalists and computational scientists.

In summary, this Research Topic elucidates a range of pertinent themes in protein structure determination, demonstrating the imperative for enhanced integration between experimental and predictive methodologies. Despite the significant advancements illustrated by AlphaFold and other AI-driven approaches, numerous challenges and opportunities remain unexplored. Future research should focus on areas such as the dynamic behavior of proteins, the role of post-translational modifications, and the interactions within protein complexes. Additionally, the development of hybrid models that seamlessly combine experimental data with AI predictions could further refine our understanding of protein structures. As the field continues to evolve, a synergistic approach will be crucial to fully realize the potential of both experimental and computational techniques in structural biology.
